# The Soft X-ray Research instrument at the Linac Coherent Light Source

**DOI:** 10.1107/S160057751500301X

**Published:** 2015-04-02

**Authors:** Georgi L. Dakovski, Philip Heimann, Michael Holmes, Oleg Krupin, Michael P. Minitti, Ankush Mitra, Stefan Moeller, Michael Rowen, William F. Schlotter, Joshua J. Turner

**Affiliations:** a Linac Coherent Light Source, SLAC National Accelerator Laboratory, 2575 Sand Hill Road, Menlo Park, CA 94025, USA; b European XFEL, Notkestrasse 85, 22607 Hamburg, Germany

**Keywords:** FEL, X-ray, ultrafast, spectroscopy, materials science

## Abstract

The Soft X-ray Research instrument provides intense ultrashort X-ray pulses in the energy range 280–2000 eV. A diverse set of experimental stations may be installed to investigate a broad range of scientific topics such as ultrafast chemistry, highly correlated materials, magnetism, surface science, and matter under extreme conditions. A brief description of the main instrument components will be given, followed by some selected scientific highlights.

## Abstract 2

A description of the Soft X-ray Research instrument (SXR) at the Linac Coherent Light Source is given. Recent scientific highlights illustrate the wide variety of experiments and detectors that can be accommodated at SXR.

## 1. Introduction   

The Soft X-ray Research instrument (SXR) was developed by a consortium to address the need for a monochromatic soft X-ray instrument at the Linac Coherent Light Source (LCLS). Members of the consortium included LCLS, Stanford Institute of Material and Energy Sciences (SIMES), the Advanced Light Source (ALS), the University of Hamburg, DESY and the Center for Free Electron Lasers (CFEL) in Hamburg.

A comprehensive discussion of all instrument components, controls and data acquisition, and initial performance can be found by Schlotter *et al.* (2012 ▶). In this paper we will briefly describe the most important instrument components and present some recent results which highlight the diverse capabilities of the SXR instrument.

## 2. Instrument overview   

**Figure 1:**
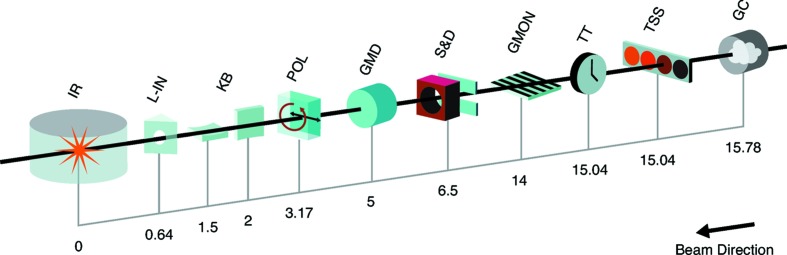
Overview of the SXR instrument layout. Distances are indicated in meters from the interaction region (IR). (GC) gas cell, (TSS) transmission sample system, (TT) timetool, (GMON) grating monochromator, (S&D) slits and diagnostics, (GMD) gas monitor detector, (POL) polarizer, (KB) Kirkpatrick–Baez mirrors, (L-IN) laser in-coupling. The sample at the SXR instrument is located approximately 176 m downstream of the undulators.

The SXR instrument spans the first two hutches in the Near Experimental Hall of the LCLS (Moeller *et al.*, 2011 ▶). A schematic of the instrument with the most important components is shown in Fig. 1 ▶. As the majority of experiments require soft X-rays precisely tuned to specific photon energies, *i.e.* usually around particular absorption edges, and with bandwidths smaller than the typical energy spread of the free-electron laser (FEL) pulses, the SXR instrument incorporates a plane-grating monochromator. Photon energy calibration is achieved by placing samples in the beam upstream of the monochromator and operating it as a spectrometer, utilizing the broad bandwidth of the self-amplified spontaneous emission (SASE) FEL pulses. Energy calibration based on absorption edges of noble gases is accomplished *via* the use of a static gas cell (GC) of length 703 mm which can be temporarily isolated from the rest of the instrument vacuum system by removable 200 nm-thick Al filters. Absorption edges of solid samples, *e.g.* 3*d* transition metals, are also used for energy calibration. This is achieved by inserting selected thin films mounted on a paddle in the transmission sample system (TSS). New thin film samples relevant for a given experiment can be loaded and ready for use in 24 h. The TSS also contains thin membranes of Si_3_N_4_ where X-ray and optical beams can intersect in space and time to provide precise shot-to-shot determination of the arrival time of the FEL pulse relative to the optical pulse (Beye *et al.*, 2012 ▶). This optical to X-ray cross-correlation system is sensitive to the changes in reflectivity induced by the X-rays (Krupin *et al.*, 2012 ▶), allowing one to correct for the inherent FEL jitter to less than 50 femtoseconds (fs) for nominal FEL and optical pulse durations of 70 and 50 fs, respectively (Beye *et al.*, 2012 ▶). This leads to an overall time resolution approaching the limit imposed only by the duration of the optical and FEL pulses. Finally, an alignment HeNe laser can be in-coupled at this location. With the monochromator operating in a non-dispersive mode, this laser propagates through all instrumental components, simulating the path of the X-ray beam; this has become a useful tool for accurate positioning of entire experimental stations and samples.

The SXR monochromator consists of two optical elements: a spherical mirror and two interchangeable plane varied-line-space gratings, ruled on the same substrate. Both optics are made from single-crystal Si, covered with B_4_C (Soufli *et al.*, 2012 ▶), and are placed at an angle of 14 mrad with respect to the incoming beam. As an instrument designed to provide high transmission, medium resolution and minimal pulse broadening of the ultrashort FEL pulses, gratings with groove density of 100 and 200 lines mm^−1^ were chosen. Calibration with neon indicates a resolving power of 2000 and 3500 for the two gratings (Heimann *et al.*, 2011 ▶), with efficiency better than 10% in first diffraction order across the entire energy range. The monochromator is designed to work without an entrance slit to avoid possible damage. The photon energy bandwidth is dispersed and focused in the vertical direction at the exit slit, where the two opposing blades can close to less than 10 µm. With the exit slits open the instrument can operate as a spectrometer by placing a YAG crystal perpendicularly to the beam 185 mm after the slits. The image is captured by a high-frame-rate optical camera, synchronized to the X-ray pulses, allowing the recording of single-shot spectra at full repetition rate of the FEL.

The inherent fluctuations both in the intensity and photon wavelength of the FEL pulses require precise non-invasive measurement of the photon flux to enable signal normalization. Immediately after the exit slit, both types of fluctuations are converted to intensity variations when the monochromator is operated in dispersive mode and a well defined wavelength is selected. At this location a gas monitor detector (GMD) is installed (Tiedtke *et al.*, 2014 ▶), where ionization of noble gas at a known pressure provides an absolute determination of the photon flux on a shot-by-shot basis. This instrument is described in more detail by Moeller *et al.* (2015 ▶).

Currently the FEL pulses at LCLS are linearly polarized in the horizontal direction; however, many experiments investigating magnetism require circularly polarized light. To modify the incident X-ray polarization, thin ferromagnetic films can be inserted into the beam and exposed to a magnetic field of 0.1 T. Angular adjustment of both the films and the magnet allows the optimization of the degree of circular polarization *via* the X-ray magnetic circular dichroism effect, well pronounced at specific resonant energies. Typical transmission through the polarizer is about 4% and the degree of circular polarization is 0.3 (Pfau *et al.*, 2010 ▶).

Focusing at the SXR instrument is accomplished with a pair of bendable Kirkpatrick–Baez mirrors. The smallest horizontal and vertical focal spot is about 2 µm × 2 µm, achieved at the same nominal focal plane, located 2 m and 1.5 m downstream of the two mirrors, respectively. The option to actively bend the curvature of the mirrors allows the beam size to be changed at the sample location; this has proven particularly useful when, for example, the highest possible overall intensity is necessary (Chalupsky *et al.*, 2011 ▶), or when a line focus is desired, as in the case of X-ray interaction with a liquid jet.

The last permanently installed instrument component is the laser in-coupling system. It consists of a set of exchangeable two-inch mirrors with holes drilled through them to allow for coaxial transmission of the X-ray beam through each mirror. This optical element is located 65 cm away from the nominal sample location, thus ensuring almost co-linear propagation of the optical and X-ray beams. Core laser systems at the LCLS consist of an ultrashort-pulsed Ti:sapphire oscillator synchronized to the FEL, seeding a commercially available chirped pulse amplifier producing 4 mJ at 40 fs. An additional home-built four-pass amplifier can boost the pulse energy to over 30 mJ to most of the experimental hutches. Wavelength conversion inside the experimental hutches can cover a broad spectral range from 200 nm to 2 THz. A more in-depth description of the optical laser capabilities at LCLS can be found by Minitti *et al.* (2015 ▶). The most important instrument specifications are summarized in Table 1 ▶.

Looking to the near future, several important projects already underway will have a significant impact on the performance and capabilities of the SXR instrument. First, the development and implementation of soft X-ray self-seeding will result in more intense narrow-band radiation. The increased photon flux will immediately benefit experiments which rely on ‘photon-hungry’ techniques such as X-ray fluorescence spectroscopy. Second, following the commissioning of the DELTA undulator currently in progress, X-rays with variable polarization, both linear vertical, horizontal and circular, will be available, thus providing a boost to experiments studying, for example, magnetism. Finally, in collaboration with the University of Connecticut, LCLS is developing a compact portable X-ray emission spectrometer with medium resolving power of 2000 at 1 keV, that could be used on a variety of endstations at LCLS, providing valuable information about inelastically scattered X-rays.

## 3. Highlights   

We present some highlights from the first few years of operation of the SXR instrument, demonstrating both the diverse set of techniques, environments and detectors that can be accommodated, and the broad range of scientific topics that can be investigated.

### 3.1. Pump–probe ultrafast chemistry   

**Figure 2:**
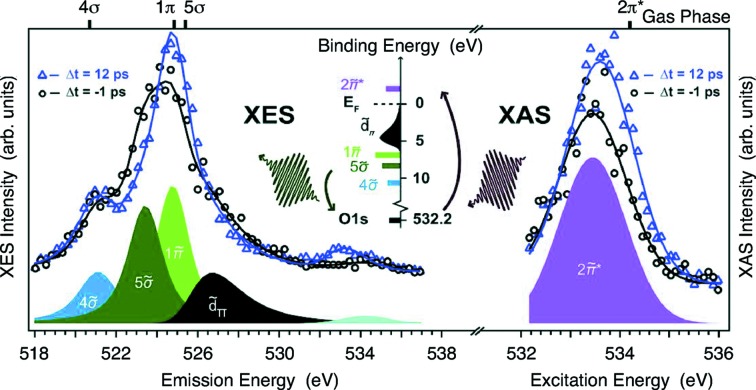
An ultrafast laser pulse heats the metal surface and initiates the process of CO desorption from the Ru surface. Snapshots of the electronic states of oxygen are captured in X-ray absorption (right) and emission (left) spectra. Figure reprinted with permission from Dell’Angela *et al.* (2013 ▶).

A fundamental problem in physical chemistry is to understand how bonds break and re-form during chemical reactions. Dell’Angela *et al.* (2013 ▶) ▶, for example, studied the dynamics of a thin surface layer of CO adsorbed on the surface of Ru(0001) after intense optical excitation using the complementary information provided by O *K*-edge absorption and emission spectroscopy, as depicted in Fig. 2 ▶. By tracing the temporal evolution of spectral features known to indicate the presence of specific chemical bonds, the researchers found out that heat generated in the Ru substrate causes the CO molecules to gain energy and ultimately desorb from the surface; however, complete breaking of the bonds is achieved only after a period of about 10 ps. In this time interval the system enters a precursor state where the molecules are weakly attached to the surface and have a finite probability of either remaining adsorbed or breaking the chemical bond and desorbing into the gas phase. This work, utilizing the surface science endstation (Katayama *et al.*, 2013 ▶), demonstrates the unique capabilities of performing spectroscopy of surface adsorbates using FELs and is an important step towards better understanding of surface chemistry and catalysis.

### 3.2. Strongly correlated materials   

Control of magnetization *via* the application of an electric field is another interesting topic, which has the potential to be exploited in the so-called multiferroic materials, where both electric and magnetic polarizations can coexist. In the next example, the speed of control of the magnetoelectric coupling is investigated by monitoring the dynamics of a magnetic diffraction peak arising from the periodic alignment of electron spins in Mn (Kubacka *et al.*, 2014 ▶).

**Figure 3:**
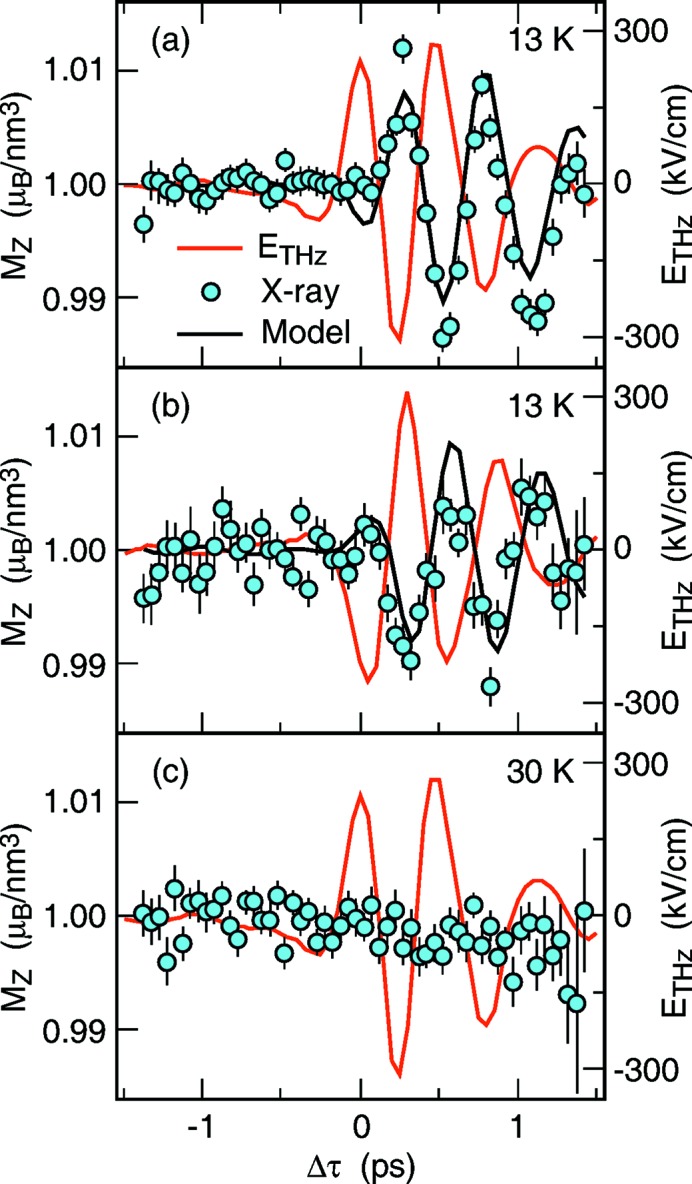
A THz pulse resonant with an electromagnon excites spin motion in the sample. An X-ray pulse resonant with the Mn *L*_2_-edge measures the response as changes in the intensity of the (0 *q* 0) diffraction peak. (*a)* Peak intensity response of the driving electric field. (*b*) Peak intensity response with opposite sign of the driving field. (*c*) The response disappears when the sample is warmed up above the spin-cycloid phase. Figure reprinted with permission from Kubacka *et al.* (2014 ▶).

TbMnO_3_ is a prototypical multiferroic, which is paramagnetic at room temperature, forms a paraelectric sinusoidal spin density wave at 42 K, and transforms into a spin-cycloid phase below 27 K. Photoexcitation with an ultrafast THz pulse, resonant with an electromagnon (electric dipole-active spin excitation) and which is present only in the spin-cycloid phase, revealed an oscillatory response of the magnetic peak, as shown in Fig. 3 ▶, with the same frequency as the driving field. This response can be attributed to the coherent rotation of the spin-cycloid plane by as much as 4°, and predicts that stronger THz pulses can rotate the magnetic polarization plane by as much as 90°. This work was carried out in the Resonant Soft X-ray Scattering endstation, which combines an in-vacuum diffractometer, cryogenic cooling capabilities and versatile configuration for laser in-coupling (Doering *et al.*, 2011 ▶; Turner *et al.*, 2015 ▶).

### 3.3. Ultrafast magnetism   

**Figure 4:**
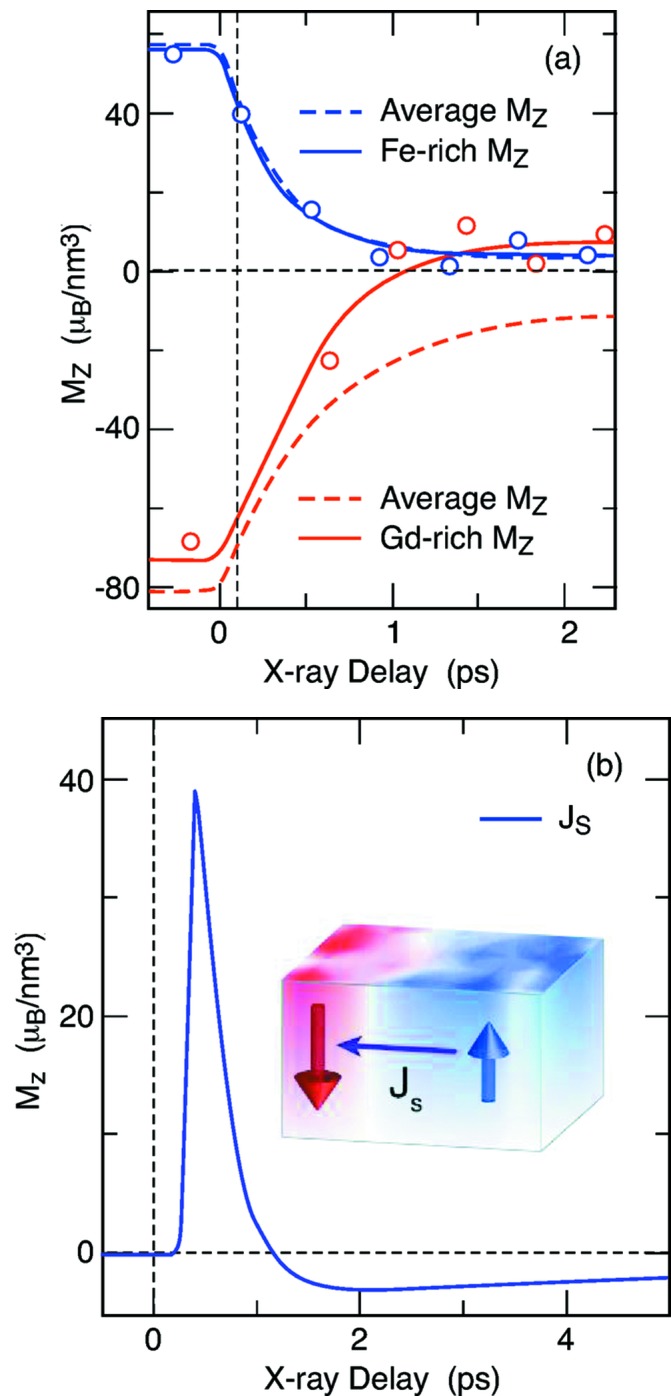
Maps of Gd_24_Fe_66.5_Co_9.5_ using scanning transmission electron microscopy with elemental sensitivity from energy-dispersive X-rays indicate the presence of nanoscale fluctuations in the iron and gadolinium concentrations. (*a*) Time evolution of the magnetization in the Gd-rich and Fe-rich regions compared with the sample average. (*b*) Time-resolved angular momentum flow into the Gd-rich regions. Figure reprinted with permission from Graves *et al.* (2013 ▶).

Another research field studied at SXR is ultrafast magnetism. Short bursts of X-rays in the soft X-ray regime can not only separate the different elements in a alloy but can also separate the response of different magnetic sub-lattices in a system. This allows investigation of how the temporal dynamics of individual nanoscale regions of a magnet behave, a question at the heart of research in magnetism, and in development for the magnetic recording industry. Graves *et al.* (2013) ▶, for example, studied the ferrimagnetic system Gd_24_Fe_66.5_Co_9.5_ and found that the time-dependence of the magnetization of both Gd and Fe can be measured independently. This brings a new understanding of the net flow of the angular momentum (Fig. 4 ▶) from one type of magnetic region to another. During the first picosecond after the excitation, the angular momentum emerges from the nanodomains of the enriched 3*d* Fe spins and flows to the 4*f* Gd-enriched magnetic regions.

### 3.4. Diagnosis of solid-density plasma   

**Figure 5:**
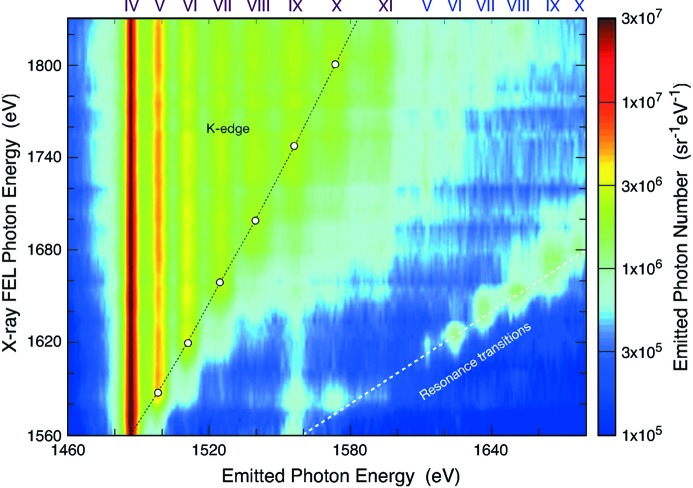
Intensity of spectrally resolved *K*α emission as a function of incident FEL excitation energy. Emission peaks from single and double *K*-shell hole are present. The results are compared with simulations incorporating radiative-collisional effects. Figure reprinted with permission from Vinko *et al.* (2012 ▶).

High energy-density matter is defined as matter that contains energy densities in the range of 10^17^ J cm^−3^. It has application to many areas, from the physics describing the center of large planets like Neptune, to fusion research. When densities reach this level, collisional effects play a critical role in determining the evolution of the charge state distribution of the solid. Vinko *et al.* (2012) ▶ recently focused 80 fs pulses down to about a 2 µm square spot size on a micrometer-thick solid aluminium sample. The focusing capability combined with high peak power results in an overall delivered intensity of order 10^17^ W cm^−2^. The photon energy was continuously tuned above the aluminium *K*-edge (1560 eV), and the charge state distribution was measured by observing the *K*-shell fluorescence from different ionic states, as presented in Fig. 5 ▶. Because the pulses are of such short duration, the sample did not undergo hydrodynamic expansion during the measurement, allowing observations of a plasma at known solid-like densities to be made. These results demonstrate that the creation of a uniform density of hot or warm dense matter becomes possible utilizing the properties of an X-ray FEL.

## 4. Conclusion   

The SXR instrument provides a versatile platform to investigate materials utilizing the unique properties of ultrafast monochromatic X-rays. The wide variety of endstations and detectors that can be accommodated allows for very flexible experimental arrangements, giving researchers the opportunity to perform experiments related to materials science, chemistry and physics. More details about the SXR instrument can be found at http://lcls.slac.stanford.edu/sxr. 

## 5. Facility access   

LCLS instruments are open to academia, industry, government agencies and research institutes worldwide for scientific investigations. There are two calls for proposals per year and an external peer-review committee evaluates proposals based on scientific merit and instrument suitability. Access is without charge for users who intend to publish their results. Prospective users are encouraged to contact instrument staff members to learn more about the science and capabilities of the facility, and opportunities for collaboration.

## Figures and Tables

**Table 1:** X-ray parameters and capabilities of the SXR instrument

Instrument name	SXR
Mirrors, incidence angle	3 B_4_C on Si, 14mrad
Monochromaticity (*E*/*E*)†	1 10^3^ (SASE), 2 10^4^ (self-seeding)
Energy range (eV)	2802000
Unfocused beam size (m)	2700 at 700eV
Focused beam size (m)	2
Focusing optics	Bendable KB (B_4_C on Si)
Flux (photons pulse^1^)	Up to 10^13^ ‡
Pulse length (fs)	5200
Repetition rate (Hz)	120, 60, 30, 10, 5, 1, on demand
Optical laser pulse energy (mJ)	20 (800nm), 45 (400nm), 1 (266nm)
Optical laser pulse width (fs)	10150
Standard detectors	pnCCD, MCP, APD, PI-MTE, Andor

† Typical single-shot value.

‡ Excluding beamline and instrument transmission.
